# Modelling of longitudinal outcomes with highly skewed distributions: applications in the IVAN trial

**DOI:** 10.1186/1745-6215-14-S1-O39

**Published:** 2013-11-29

**Authors:** R Nash, L Scott, U Chakravarthy, SP Harding, BC Reeves, CA Rogers

**Affiliations:** 1 Clinical Trials and Evaluation Unit, School of Clinical Sciences, University of Bristol, Bristol, UK; 2 Institute of Clinical Science, The Queen’s University of Belfast, Belfast, UK; 3 Department of Eye and Vision Science, Institute of Ageing and Chronic Disease, University of Liverpool, Liverpool, UK

## 

The IVAN trial is a multi-centre factorial non-inferiority randomised controlled trial of 610 participants to compare treatments for neovascular Age-related Macular Degeneration (two drugs, Lucentis and Avastin, and two treatment regimens, treatment monthly and treatment as-needed). Many of the trial outcomes are longitudinal continuous outcomes measured at regular intervals over the two-year study period.

The aim of the analyses was to obtain estimates of the drug and treatment regimen effects at two-years. For many of the outcomes, standard mixed-effects models fitted the data well. However, for some secondary outcomes, and in particular total lesion area, the distributions were highly skewed; 28% of 1536 measurements of lesion area were zero indicating a lesion was not present, which rendered standard regression modelling unsuitable.

One approach taken to analyse this outcome was to dichotomise the variable into lesion present and lesion absent, and fit a logistic regression model with repeated measures. This analysis identified a statistically significant difference between the proportions of patients with no lesion present at two-years on the different treatment regimens. However the analysis did not make full use of the data available and information about the size of the lesion was not evaluated.

An alternative approach that we have investigated is to use two-part modelling whereby the dichotomised data is analysed using logistic regression, and the non-zero values then analysed using mixed-effects models. We will present the results of this analysis and describe the methodological challenges faced when implementing it in the context of the IVAN trial.

